# Nanomaterials and Textiles

**DOI:** 10.3390/nano14231900

**Published:** 2024-11-27

**Authors:** Boris Mahltig, Andrea Ehrmann

**Affiliations:** 1 Faculty of Textile and Clothing Technology, Hochschule Niederrhein, University of Applied Sciences, 41065 Mönchengladbach, Germany; 2 Faculty of Engineering and Mathematics, Bielefeld University of Applied Sciences and Arts, 33619 Bielefeld, Germany

## 1. Introduction

**Figure 1:**
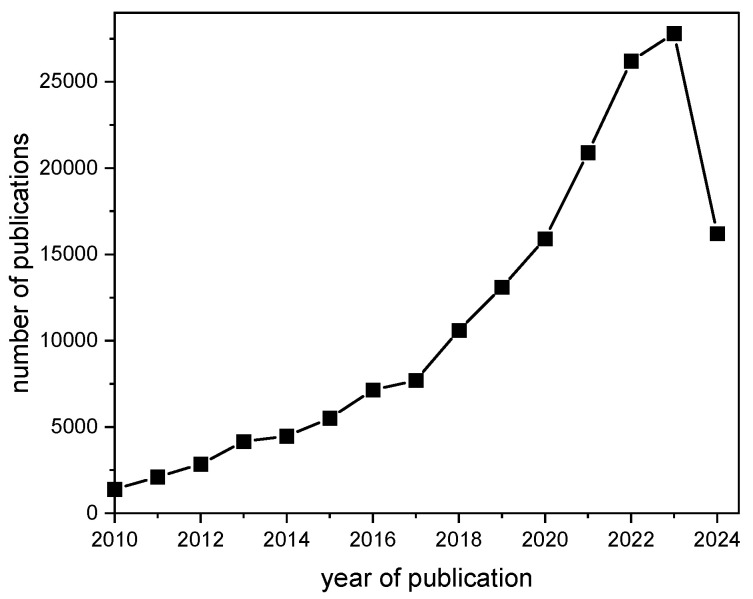
The number of publications containing both the terms “nanomaterials” and “textiles” found in a Google Scholar search performed in October 2024. The total number of publications found was around 207,000.

The terms “nano” and “nanomaterials” have come to be buzz words describing tremendous advances in research and development during the last decades [1,2,3,4]. In comparison, textiles—or, in general, conventional fiber-based materials—have been used for thousands of years and can arguably be considered traditional materials [5,6]. The creation of new functional products by the addition of nanomaterials has come to constitute an interesting development in research [7,8], with this including textile products gaining enhanced properties after the application of nanomaterials [9,10,11]. This major development in the field is reflected by the high number of scientific publications containing both the terms “nanomaterials” and “textiles”. A search on Google Scholar performed in October 2024 detected a high number of approximately 207,000 publications in this area. Furthermore, remarkable growth in the number of publications related to nanomaterials and textiles can be observed within the last 15 years (Figure 1). However, this research is not only related to new textile products but also to disadvantageous effects on health caused by nanomaterials in combination with textiles or released from textiles [12,13]. A prominent issue in this field of research is the release of so-called “nanoplastics” from textile products [14,15].

To address the growing interest in this emerging field of development, this Special Issue presents an overview of a broad range of different nanomaterials and the advantages of applying them to textile-based materials for the creation of new materials with advanced or completely new properties. Prominent examples in this field include nanoparticular (sol–gel-based) finishing agents for antimicrobial or flame-retardant functionalization [16,17,18,19]; the embedding of particles into fibers during spinning processes, which can be used to realize textile materials with radiation-protective properties [20,21,22,23]; and phosphorescence inorganic particles or fluorescent carbon quantum dots applied on textiles, resulting in materials with luminescent effects [24,25,26,27]. Additionally, a broad field for nanomaterials and textiles is that of electrospinning, affording the opportunity of, e.g., creating new functional filter materials [28,29,30,31].

## 2. Overview of Published Articles

To address the different aspects of nanomaterials and textiles, this Special Issue presents the following articles, which cover a broad range of different materials and their applications.

Among the review papers, Malucelli (contribution 1) provides an overview of nanostructured flame-retardant layers on cotton fabrics, and Bencurova et al. (contribution 2) discuss the possibility to produce organic chips and nanocellulose-based transistors in particular.

Several authors report novel experimental findings. Tan et al. (contribution 3) investigated the electrochemical performance of coal-derived carbon nanofibers, whereas Munir et al. (contribution 4) developed carbon-nanodot-loaded PLA nanofibers for medical applications. Dissanayake et al. (contribution 5) tested dye removal by a functionalized nanofiber membrane. Reinforcing textiles with electrospun nanofibers was a solution suggested by Sanchaniya et al. (contribution 6) to avoid crimping. Morina et al. (contribution 7) discussed the inhomogeneities of nanofiber mats, whereas Pakolpakcil et al. (contribution 8) optimized centrifugal spinning parameters, and Liu et al. (contribution 9) investigated the homogeneity of the electric field in radial multi-nozzle electrospinning. Filaments loaded with luminescent nanoparticles were examined by Yust et al. (contribution 10), whereas Kanamori et al. (contribution 11) developed poly (vinyl alcohol) nanofibers for drug delivery, and Dragar et al. (contribution 12) investigated both the influence of incorporated drugs and the properties of hydrophilic nanofibers.

## 3. Conclusions

In summary, this Special Issue presents comprehensive research outlining the progress made in the field of nanomaterials and textiles, with a focus on the application of nanomaterials to improve the performance of textiles or even initiate new functional properties. Nevertheless, due to the broadness of this field, it is nearly impossible to cover all relevant aspects, and, for this reason, a second volume for this Special Issue will be considered.
